# Integrating Lymph Node Metastasis and Programmed Death-Ligand 1 Prediction in Non–Small Cell Lung Cancer From a Single PET/CT Scan: Multicenter Radiomics Study

**DOI:** 10.2196/86835

**Published:** 2026-09-09

**Authors:** Wen Chen, Qiufang Liu, Huiling Peng, Jianping Zhang, Zhihao Chen, Silong Hu, Shaoli Song

**Affiliations:** 1 Department of Nuclear Medicine, Fudan University Shanghai Cancer Center, Fudan University, Rd. Dongan 270, Shanghai, Shanghai, 2000027, China, 86 68388096, 86 68388096; 2 College of Biomedical Engineering, Yiwu Research Institute, Fudan University, Shanghai, Shanghai, China; 3 Department of Oncology, Shanghai Medical College, Fudan University, Shanghai, Shanghai, China; 4 Center for Biomedical Imaging, Fudan University, Shanghai, Shanghai, China; 5 Key Laboratory of Nuclear Physics and Ion-beam Application (MOE), Fudan University, Shanghai, Shanghai, China; 6 Department of Nuclear Medicine, Shanghai Proton and Heavy Ion Center, Fudan University Cancer Hospital, Shanghai, Shanghai, China; 7 Department of Research and Development, Shanghai United Imaging Intelligence Company Ltd, Shanghai, Shanghai, China

**Keywords:** non–small cell lung cancer, NSCLC, positron emission tomography, PET, computed tomography, CT, PET/CT, radiomics, machine learning, peritumoral region, programmed death-ligand 1, PD-L1 expression

## Abstract

**Background:**

Preoperative stratification for non–small cell lung cancer (NSCLC) necessitates separate evaluations of lymph node metastasis (LNM) to guide surgical decisions and of programmed death-ligand 1 (PD-L1) expression to inform immunotherapy.

**Objective:**

This study aimed to develop and validate an integrated diagnostic solution that could simultaneously predict both LNM status and PD-L1 expression status from a single, standard-of-care ^18^F-fluoro-2-deoxy-D-glucose positron emission tomography/computed tomography (^18^F-FDG PET/CT) scan.

**Methods:**

In this multicenter study, we segmented primary tumors and peritumoral 15-mm expansion regions from preoperative PET/CT scans of 273 patients (for LNM prediction) and 242 patients (for PD-L1 prediction). A total of 7868 radiomic features from intratumoral and peritumoral regions were extracted. Following rigorous feature selection, 2 independent models were developed using machine learning and tested on a temporal validation cohort (n=45). Model performance was benchmarked against clinicopathological models and nuclear medicine physicians.

**Results:**

The integrated model for LNM prediction (PT-IPT-LR) achieved an area under the curve of 0.845 (95% CI 0.716‐0.973) in the temporal validation cohort, with a sensitivity of 0.765 (95% CI 0.518‐1.000) and a specificity of 0.786 (95% CI 0.602‐0.970). The model for PD-L1 expression (PT-IPT-SVM) achieved an area under the curve of 0.776 (95% CI 0.641‐0.911) in the temporal validation cohort, with a sensitivity of 0.800 (95% CI 0.609‐0.991) and a specificity of 0.650 (95% CI 0.401‐0.899). Decision curve analysis confirmed the clinical utility of both models. Critically, we found no significant correlation between the radiomic signatures of LNM and PD-L1, which validates our 2-model approach.

**Conclusions:**

We present a radiomics framework that noninvasively integrates prediction of LNM and PD-L1 from a single preoperative PET/CT scan. This tool may enable preoperative stratification, potentially optimizing both surgical and systemic treatment planning for patients with NSCLC in a single step.

## Introduction

Non–small cell lung cancer (NSCLC) accounts for approximately 80% to 85% of lung cancers and remains the leading cause of cancer-related mortality worldwide [1]. An accurate tumor node metastasis (TNM) staging system is critical for prognostication and individualized treatment planning. According to clinical guidelines, radical surgical resection is the standard of care for early-stage NSCLC (T1-3N0-1M0), whereas patients with advanced-stage disease receive systemic therapies such as chemotherapy, immunotherapy, or targeted therapy [2]. Therefore, preoperative staging is indispensable for therapeutic decision-making.

In clinical practice, 2 distinct diagnostic assessments are paramount for optimizing this decision-making process. First, surgical management hinges on accurate lymph node (LN) status. Surgery is contraindicated for patients with bulky or multistation N2 disease, whereas systematic nodal dissection during resection for early-stage NSCLC [3] increases the risk of complications without survival benefit for those without mediastinal involvement [4]. The optimal lymphadenectomy strategy remains controversial [5], underscoring the urgent need for reliable noninvasive preoperative LN assessment. Conventional imaging modalities, including computed tomography (CT) and ^18^F-fluoro-2-deoxy-D-glucose positron emission tomography/computed tomography (^18^F-FDG PET/CT), exhibit suboptimal diagnostic performance. CT demonstrates sensitivity and specificity of 71.2%‐73.2% and 67.2%‐79.3%, respectively, for mediastinal lymph node metastasis (LNM) [6,7], with up to 60% of cN1 cases upgraded to N2 disease postthoracotomy [8]. PET/CT shows variable sensitivity (48.5%‐81.3%) and specificity (83.0%‐87.7%) [9,10]. Overall, these limitations necessitate novel noninvasive diagnostic approaches. Second, immune checkpoint inhibitors targeting the programmed cell death protein 1 and programmed death-ligand 1 (PD-L1) axis significantly improve outcomes [11,12]. PD-L1 tumor proportion score (TPS), assessed via immunohistochemistry (IHC) on tissue specimens, is a validated predictive biomarker. However, IHC-based detection has inherent limitations, including tumor heterogeneity, spatial sampling bias, invasive tissue acquisition, and the inability to dynamically monitor expression during treatment.

Consequently, the current preoperative workflow is inherently fragmented, relying on separate and often invasive procedures to answer 2 critical but distinct questions: “Is the patient a candidate for curative surgery?” and “Is the patient a candidate for immunotherapy?” This disconnect highlights a pressing clinical need for an integrative, noninvasive tool that can simultaneously evaluate both locoregional spread and systemic therapy eligibility.

Radiomics, the high-throughput extraction of quantitative imaging features, has emerged as a promising solution for noninvasive tumor characterization [13]. While previous studies have demonstrated the potential of PET/CT radiomics for predicting epidermal growth factor receptor (EGFR) mutations [14,15], LNM [16,17], as well as for exploring correlations with PD-L1 expression [18-21], significant gaps remain. Most approaches are single-task-oriented, focusing either on LNM or PD-L1, thereby failing to address the integrated clinical need. Furthermore, many studies have primarily focused on intratumoral features, overlooking the peritumoral region, which harbors critical information about the tumor microenvironment and immune response [22,23]. Critically, the relationships between preoperative LNM status, PD-L1 expression, and their respective radiomic signatures remain largely unexplored, a knowledge gap that is pivotal for developing effective dual-target prediction models.

This study aimed to develop and validate an integrative radiomics framework using preoperative ^18^F-FDG PET/CT for the simultaneous and independent prediction of LNM (task A) and PD-L1 expression status (task B) in NSCLC. Our specific objectives were to: (1) assemble a 2-center cohort with diverse imaging protocols; (2) extract high-throughput radiomic features from both intratumoral and peritumoral regions (15-mm expansion) across PET and CT modalities; (3) develop and validate 2 dedicated prediction models through a machine learning pipeline; (4) benchmark model performance against clinicopathological models and nuclear medicine physicians; and (5) investigate the correlation between the radiomic features predictive of LNM and those predictive of PD-L1 expression to elucidate their biological interplay.

## Methods

### Ethical Considerations

This retrospective study was conducted in accordance with the principles of the Declaration of Helsinki (revised in 2013) and relevant institutional guidelines and regulations. The study was approved by the Institutional Ethics Committee of the Fudan University Shanghai Cancer Center and the Shanghai Heavy Ion Hospital (1909207-14-1910 and 200217EXP-01). Written informed consent was waived because of the retrospective nature of the study. To ensure privacy, all images and relevant data were deidentified before analysis and reporting, with no personally identifiable information included. Participants did not receive financial or other material compensation for their involvement.

### Study Design

**Figure 1.**
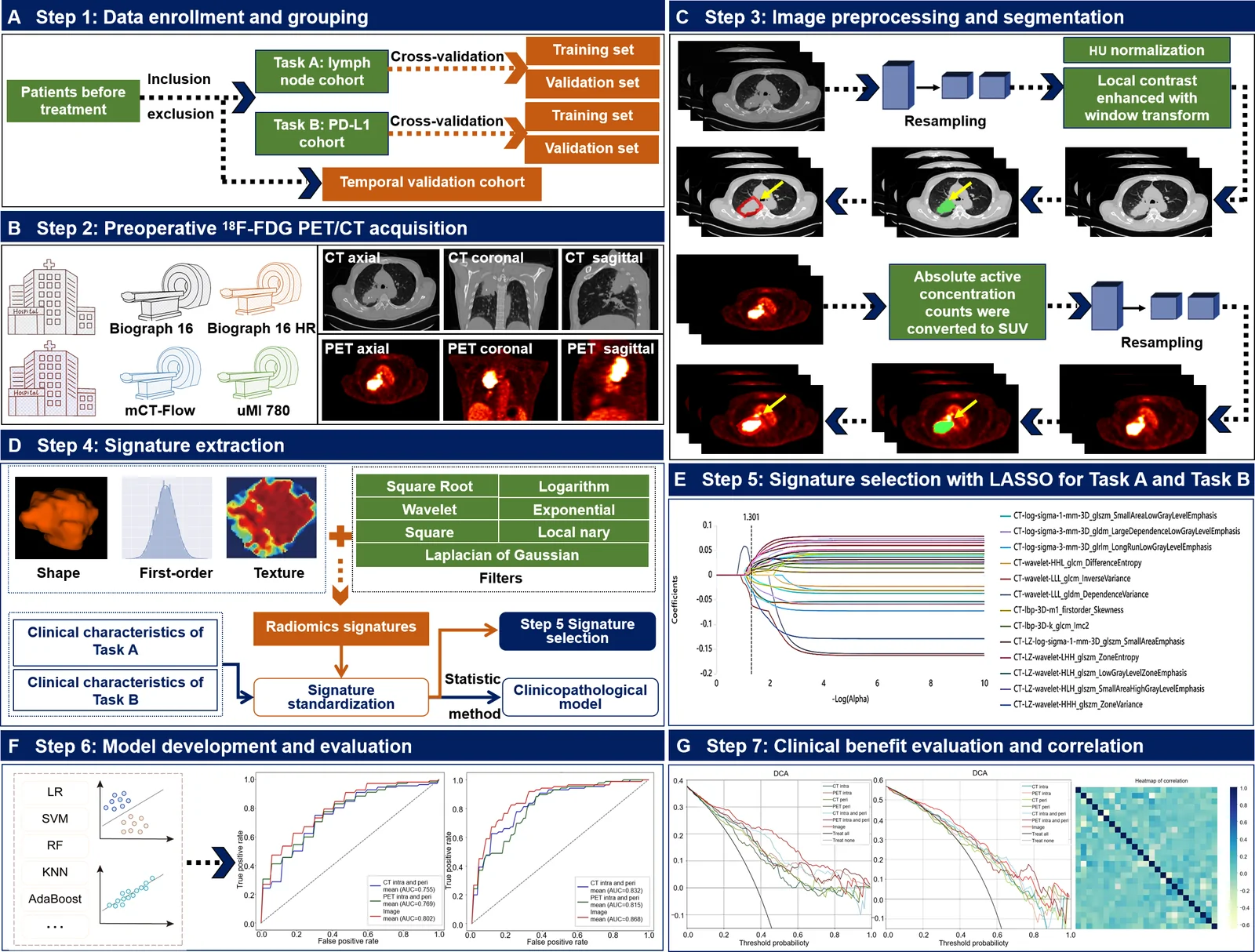
Workflow of preoperative ^18^F-FDG PET/CT radiomics in patients: prediction of lymph node metastasis and programmed death-ligand 1 (PD-L1) expression. The process consists of 7 core steps: (A) patient data enrollment and grouping, (B) preoperative ^18^F-FDG PET/CT imaging acquisition, (C) image preprocessing and segmentation, (D) signature extraction, (E) LASSO signature selecting, (F) development and validation of multiple models, and finally, (G) clinical value assessment through decision curve analysis (DCA). AdaBoost: adaptive boosting; DCA: decision curve analysis; HU: Hounsfield unit; KNN: k-nearest neighbors; LASSO: least absolute shrinkage and selection operator; LR: logistic regression; RF: random forest; SUV: standardized uptake value; SVM: support vector machine; ^18^F-FDG PET/CT: ^18^F-fluoro-2-deoxy-D-glucose positron emission tomography/computed tomography.

The radiomics workflow for predicting LNM and PD-L1, as depicted in Figure 1, consists of 7 steps: patient enrollment and stratification; acquisition of preoperative ^18^F-FDG PET/CT; preprocessing and segmentation for both imaging modalities; extraction of radiomics features, along with clinical variables, followed by standardization; feature selection using least absolute shrinkage and selection operator (LASSO) regression for both prediction tasks; development of machine learning models and evaluation using appropriate metrics; and assessment of clinical utility through decision curve analysis (DCA), as well as exploration of feature correlations.

### Patient Selection

The clinical data of participants who underwent surgical resection and PD-L1 TPS testing between February 2016 and February 2024 were retrospectively analyzed. Pathological evaluations were performed by specialized pathologists, who recorded the following: (1) tumor size, invasion, and lymph node involvement according to the *American Joint Committee on Cancer TNM Staging Manual*, Eighth Edition; (2) differentiation grade; (3) lymphovascular space invasion; (4) perineural invasion. PD-L1 IHC staining was conducted on surgical specimens. TPS was calculated as the percentage of PD-L1 positive tumor cells among all viable tumor cells, with positivity defined as ≥1% staining [24]. All IHC analyses were performed by pathologists at 2 hospitals.

**Figure 2.**
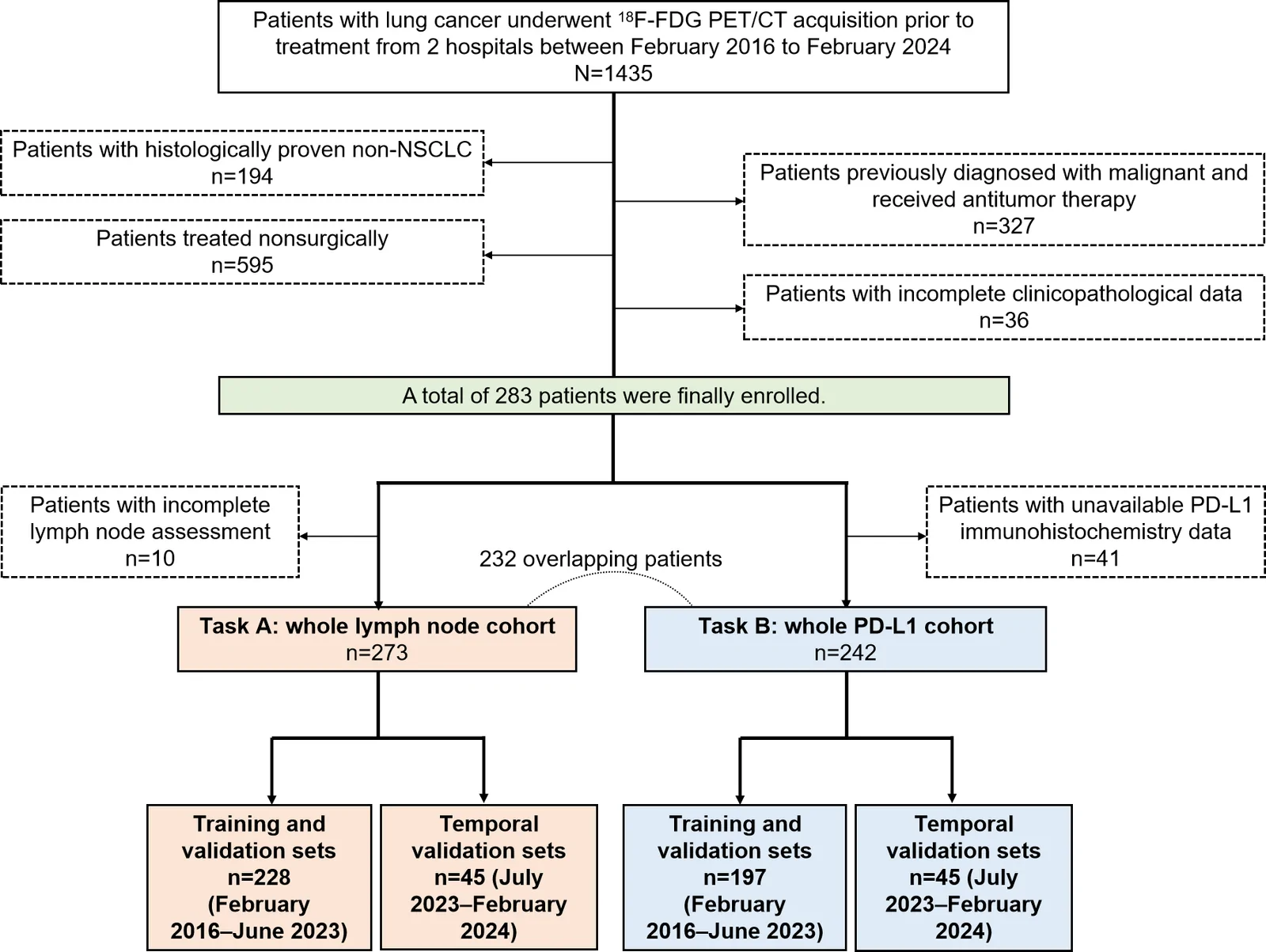
The flowchart of inclusion or exclusion and cohort grouping for patients with lung cancer at the 2 hospitals. IHC: immunohistochemistry; NSCLC: non–small cell lung cancer; PD-L1: programmed cell death ligand 1; PET/CT: positron emission tomography/computed tomography.

The inclusion criteria were as follows: (1) aged 18 years or older; (2) histologically confirmed NSCLC; (3) all participants had undergone curative-intent surgical resection of the primary lung tumor and hilar and/or mediastinal lymphadenectomy; (4) complete clinical data (sex, age, tumor size, tumor markers, and TNM stage); (5) available ^18^F-FDG PET/CT scan data before treatment. The exclusion criteria were as follows: (1) incomplete clinicopathological data, (2) concurrent malignancies, and (3) preoperative antitumor therapy (Figure 2). A total of 283 patients were recruited from 2 centers from February 2016 to February 2024. Two independent but partially overlapping analytical cohorts were delineated based on the availability of data for specific predictive endpoints. The LNM cohort (n=273) was established by excluding 10 patients with incomplete lymph node assessment, and the PD-L1 cohort (n=242) was formed by excluding 41 patients with unavailable PD-L1 IHC data. Both cohorts were stratified according to enrollment time into training and validation sets (from February 2016 to June 2023) and independent temporal validation sets (n=45 each, from July 2023 to February 2024). There were 232 overlapping participants (82.0% of the total cohort) who had complete data for both LNM and PD-L1. The training sets for task A (n=228) and task B (n=197) overlapped by 187 patients, while the temporal validation sets for both tasks consisted of the same 45 patients. For the diagnosis of lymph node metastasis, a lymph node was defined as positive if it exhibited a short-axis diameter greater than 10 mm and/or ^18^F-FDG uptake exceeding that of the mediastinal blood pool. Nuclear medicine physicians performed their assessment under blinded conditions, with access only to the preoperative PET/CT images and without any additional clinical data. For patient-level diagnosis, the presence of any single lymph node meeting the positivity criteria was sufficient to classify the patient as node-positive. As there is no established or standardized clinical method for a nuclear medicine physician to visually or semiquantitatively estimate PD-L1 expression from an ^18^F-FDG PET/CT scan, we chose to compare our radiomics model against the robust clinicopathological benchmark currently used in clinical practice.

### Preoperative PET/CT Acquisition

PET/CT scans were performed using Siemens Biograph 16, Biograph 16 HR, Biograph mCT-Flow (Siemens Healthineers), and United Imaging Healthcare (UIH) uMI 780 systems. Patients fasted ≥6 hours pre-scan, with blood glucose smaller than 140 mg/dL. ^18^F-FDG at a dose of 3.7 MBq/kg (0.1 mCi/kg) was injected intravenously, and PET/CT imaging (skull to midthigh) commenced 60 minutes postinjection.

For the Siemens Biograph mCT-Flow, a helical CT scan at 120 kV, 140 mA, and 3 mm slice thickness was performed. The PET scan (2 min/bed) was reconstructed using the ordered-subsets expectation maximization (OSEM) algorithm with CT attenuation correction. For the Siemens Biograph 16/16HR, a low-dose CT scan was first performed (120 kV and 150 mA for the Biograph 16; 120 kV and 140 mA for the Biograph 16HR). The PET scan (2‐3 min/bed) was reconstructed using a Gaussian-filtered iterative reconstruction algorithm (4 iterations, 8 subsets). For the UIH uMI 780, CT scanning was first performed at 120 kV with auto-mAs (15‐100 mA). PET data (6‐8 beds, 35% overlap) were subsequently reconstructed using OSEM (2 iterations, 20 subsets).

### Image Preprocessing and Segmentation

DICOM-formatted PET/CT images were exported from picture archiving and communication systems. CT images (resolution 512×512; voxel size 0.59‐1.37×0.59‐1.37×5 mm³) were resampled to 1×1×1 mm³ via nearest-neighbor interpolation, normalized to [–1000, 400] Hounsfield unit [25], and window-transformed to enhance tumor visibility. PET images (resolution 168‐200×168‐200; voxel size 4.06‐4.07×4.06‐4.07×4‐5 mm³) were converted to standardized uptake values (SUVs) [26], discretized, and identically resampled. This preprocessing pipeline was implemented using PyCharm (v2018.2, JetBrains) to enhance the texture discrimination capability of the images and improve the similarity and robustness of radiomic features derived from different scanning equipment and protocols.

Primary tumors were delineated by 2 blinded nuclear medicine physicians using ITK-SNAP (v4.0.1). Interobserver agreement was quantified via the intraclass correlation coefficient (ICC) [27]. Peritumoral regions were automatically generated by expanding by 15 mm [28-30] using PyCharm. We applied an automatic segmentation algorithm with lung parenchyma restriction and interference structure exclusion rules to eliminate nontumor structures and generate clinically applicable tumor-peripheral regions of interest. Subsequent radiomic feature selection further filtered out residual noisy features resulting from anatomical contamination, ensuring the robustness of the signatures for predicting NSCLC lymph node metastasis and PD-L1 expression.

### Feature Extraction

Quantitative radiomics features were extracted from intratumoral and peritumoral regions from PET and CT using PyCharm. These features were categorized into 4 groups: first-order statistics (eg, variance, skewness, and kurtosis); shape features (volume, surface area, and sphericity); texture features (gray-level cooccurrence matrix, gray-level run length matrix, gray-level size zone matrix, and gray-level dependence matrix); filtered features (wavelet, Laplacian of Gaussian, square, logarithmic, and exponential filters) [31-33].

### Feature Selection

Radiomic features were normalized using a *z*-score transformation on the uRP workstation [34]. Clinical variables used for the clinicopathological benchmark model were standardized in the same way, but were not included in the radiomic feature set. To prevent data leakage, normalization parameters for the radiomic features were derived solely from the training cohort before being applied to both training and test sets. A tailored multistep feature selection strategy, which included variance thresholding, correlation filtering, and the application of LASSO regression, was used for different data modalities and prediction tasks on the same workstation. The detailed workflow is illustrated in Figure S1 in Multimedia Appendix 1.

Briefly, for single-modality analysis, features were selected separately from 3 regions of interest (intratumoral, peritumoral, and combined intratumoral and peritumoral) on the uRP, yielding 6 optimal feature subsets. For dual-modality modeling in task A, these 6 subsets were merged, and a secondary feature selection step was performed on the workstation to identify the final feature subset. In contrast, for task B, the final feature subset was selected directly from the entire raw feature pool to circumvent potential information loss from preliminary screening. Heatmaps were generated to visualize feature correlations. Predictors for clinicopathological models were screened using univariate logistic regression (*P*<.05) [35] and LASSO on the same workstation.

### Model Development and Evaluation

Using the uRP workstation, the selected radiomics features were used to train 11 diverse machine learning classifiers, encompassing a wide range of algorithms (eg, tree-based methods like random forest, linear models like logistic regression, and kernel-based methods such as support vector machines). Model optimization was performed on the workstation using 5-fold cross-validation with hyperparameter tuning. All final selected hyperparameters for all models are shown in Multimedia Appendix 1.

To prevent leakage, cohorts were separated first, and all model development steps used only the training cohort. The temporal testing cohort was evaluated once with the final model. Model performance was evaluated on the uRP through 2 validation strategies: internal validation, which was conducted using the training and validation cohorts, and temporal testing, which was implemented using an independent test cohort. Metrics included receiver operating characteristic (ROC) curves, area under the curve (AUC) with 95% CIs, sensitivity, specificity, accuracy, precision, and *F*_1_-score. Clinical utility was assessed via DCA on the same platform.

### Statistical Analysis

Intergroup comparisons used 2-tailed independent sample *t* tests (normally distributed continuous variables), Mann-Whitney *U* tests (nonnormal continuous variables), and Fisher exact and chi-square tests (categorical variables). Univariate logistic regression identified predictors for target tasks. Interobserver segmentation agreement was calculated via ICC. The *z* test was used to compare the differences in AUC between subgroups. The optimal operating threshold for binary classification was determined using the maximum Youden index, which was calculated exclusively on the training cohort during 5-fold cross-validation. This prespecified threshold was then applied unchanged to the independent temporal testing cohort to avoid data leakage and ensure unbiased performance estimation.

Scanner subgroup analysis was performed exclusively on the independent temporal testing cohort to assess model generalization across different imaging systems. Between-group comparisons were conducted using the *z* test for independent AUC estimates. A 2-sided *P*<.05 was regarded as statistically significant.

## Results

### Study Cohort Characteristics

As shown in Figure 2, the final merged cohort comprised 283 participants. Two analytical cohorts were established for the respective predictive tasks. The LNM cohort (n=273) included patients with complete lymph node pathological evaluation, while the PD-L1 cohort (n=242) consisted of those with available PD-L1 IHC results and TPS assessment. A total of 232 participants overlapped between the 2 cohorts, due to the concurrent availability of both sets. Both cohorts were partitioned into training and validation sets (February 2016-June 2023) and temporal validation sets (n=45 each, July 2023-February 2024). Clinicopathological characteristics of the LNM and PD-L1 cohorts are summarized in Table 1 and Table 2, respectively. The clinical characteristics of the temporal validation cohort are summarized in Table S1 in Multimedia Appendix 1.

**Table 1.** Clinical characteristics of task A in the training and validation sets. The *P* values represent the comparison between groups.

Clinical characteristics	Training and validation			
	All	No LNM^a^	LNM	*P* value
Total, n (%)	228 (100)	142 (62.3)	86 (37.7)	
Age (y)				.11
Mean *±* SD (range)	61.8 *±* 5.9 (27-81)	62.5 *±* 5.5 (27-81)	60.6 *±* 6.3 (37-79)	
Sex, n (%)				.68
Female	78 (34.2)	50 (35.2)	28 (32.6)	
Male	150 (65.8)	92 (64.8)	58 (67.4)	
Histopathology, n (%)				.04
Squamous cell carcinoma	67 (29.4)	46 (32.4)	21 (24.4)	
Adenocarcinoma	149 (65.4)	91 (64.1)	58 (67.4)	
Others	12 (5.3)	5 (3.5)	7 (8.1)	
Grade of histology, n (%)				.003
Poorly	88 (38.6)	43 (30.3)	45 (52.3)	
Moderately-poorly	69 (30.3)	43 (30.3)	26 (30.2)	
Moderately	58 (25.4)	46 (32.4)	12 (14.0)	
Highly-moderately	5 (2.2)	5 (3.5)	0 (0)	
Highly	2 (0.9)	2 (1.4)	0 (0)	
Unknown	6 (2.6)	3 (2.1)	3 (3.5)	
Primary tumor location, n (%)				.57
Right upper lobe	57 (25.0)	34 (23.9)	23 (26.7)	
Left upper lobe	63 (27.6)	38 (26.8)	25 (29.1)	
Left lower lobe	39 (17.1)	28 (19.7)	11 (12.8)	
Right lower lobe	53 (23.3)	30 (21.1)	23 (26.7)	
Right middle lobe	16 (7.0)	12 (8.4)	4 (4.7)	
Imaging classification, n (%)				.05
Central	53 (23.3)	29 (20.4)	24 (27.9)	
Peripheral	173 (75.9)	113 (79.6)	60 (69.8)	
Central with peripheral	2 (0.9)	0 (0)	2 (2.3)	
Visceral pleural invasion, n (%)				.002
Yes	44 (19.3)	19 (13.4)	25 (29.1)	
No	176 (77.2)	120 (84.5)	56 (65.1)	
Unknown	8 (3.5)	3 (2.1)	5 (5.8)	
Volume of tumor (cm^3^)				.45
Median (range)	11.1 (0.7‐592.1)	7.8 (0.7‐592.1)	19.9 (0.8‐203.0)	
SUV^b^_max_				.01
Median (range)	9.7 (0.1‐39.5)	7.6 (0.1‐39.5)	10.9 (0.3‐32.2)	
SUV_mean_				.003
Median (range)	3.7 (0.0‐14.0)	3.0 (0.0‐14.0)	4.4 (0.2‐12.6)	
SUV_median_				.003
Median (range)	4.8 (0.1‐136.5)	3.7 (0.1‐129.0)	8.9 (0.3‐136.5)	
SUV_min_				.56
Median (range)	0.5 (0.0‐1.4)	0.5 (0.0‐1.2)	0.5 (0.1‐1.4)	
2D diameter of tumor (cm)				.02
Median (range)	3.9 (1.3‐12.8)	3.4 (1.3‐12.8)	4.5 (1.5‐8.8)	
3D diameter of tumor (cm)				.03
Median (range)	4.3 (1.6‐15.4)	3.8 (1.6‐15.4)	5.1 (1.7‐10.4)	
MTV^c^ (cm^3^)				.047
Median (range)	4.8 (0.1‐136.5)	3.7 (0.1‐129.0)	8.9 (0.3‐136.5)	
TLG^d^				.13
Median (range)	15.8 (0.0‐1734.5)	10.1 (0.0‐1734.5)	30.2 (0.5‐1120.0)	
Heterogeneity factor				.31
Median (range)	2.2 (0.5‐12.6)	2.2 (0.5‐12.6)	2.3 (0.9‐7.0)	
CEA^e^				.20
Median (range)	3.0 (0.5‐144)	2.9 (0.8‐118)	3.3 (0.5‐144)	
CYFRA21-1^f^				.49
Median (range)	2.9 (0.9‐189.0)	2.8 (0.9‐189.0)	3.3 (1.2‐78.1)	
NSE^g^				.05
Median (range)	12 (7.0‐63.9)	11.9 (7.0‐39.5)	12.2 (7.0‐63.9)	

a LNM: lymph node metastasis.

b SUV: standardized uptake values.

c MTV: metabolic tumor volume.

d TLG: total lesion glucose.

e CEA: carcinoembryonic antigen.

f CYFRA21-1: cytokeratin 19 fragment‌.

g NSE: neuron-specific enolase.

**Table 2.** Clinical characteristics of task B in training and validation sets. The *P* values represent the comparison between groups^a^.

Clinical characteristic	Training and validation			
	All	Negative	Positive	*P* value
Total, n (%)	197 (100)	85 (43.1)	112 (56.9)	
Age (y)				.59
Mean *±* SD (range)	62.5 *±* 8.6 (39-80)	63 *±* 9.5 (41‐77)	62.1 *±* 8.0 (39-80)	
Sex, n (%)				.02
Female	70 (35.5)	38 (44.7)	32 (28.6)	
Male	127 (64.5)	47 (55.3)	80 (71.4)	
Histopathology, n (%)				.31
Squamous cell carcinoma	60 (30.5)	23 (27.1)	37 (33.0)	
Adenocarcinoma	132 (67.0)	61 (71.8)	71 (63.4)	
Others	5 (2.5)	1 (1.2)	4 (3.6)	
Grade of histology, n (%)				.13
Poorly	69 (35.0)	25 (29.4)	44 (39.3)	
Moderately-poorly	68 (34.5)	27 (31.8)	41 (36.6)	
Moderately	51 (25.9)	28 (32.9)	23 (20.5)	
Highly-moderately	7 (3.6)	4 (4.7)	3 (2.7)	
Highly	2 (1.0)	1 (1.2)	1 (0.9)	
Primary tumor location, n (%)				.67
Right upper lobe	56 (28.4)	27 (31.8)	29 (25.9)	
Left upper lobe	57 (28.9)	23 (27.1)	34 (30.4)	
Left lower lobe	29 (14.7)	10 (11.8)	19 (17.0)	
Right lower lobe	44 (22.3)	19 (22.4)	25 (22.3)	
Right middle lobe	11 (5.6)	6 (7.1)	5 (4.5)	
Imaging classification, n (%)				.39
Central	42 (21.3)	15 (17.6)	27 (24.1)	
Peripheral	153 (77.7)	68 (80.0)	85 (75.9)	
Central with peripheral	2 (1.0)	2 (2.4)	0 (0)	
Visceral pleural invasion, n (%)				.02
Yes	35 (17.8)	9 (10.6)	26 (23.2)	
No	154 (78.2)	74 (87.1)	80 (71.4)	
Unknown	8 (4.1)	2 (2.4)	6 (5.4)	
Volume of tumor (cm^3^)				.27
Median (range)	10.2 (0.7‐227.8)	9.1 (0.9‐166.4)	11.9 (0.7‐227.8)	
SUV^b^_max_				.007
Median (range)	9.6 (0.3‐51.7)	6.4 (0.3‐24.8)	10.5 (0.8‐51.7)	
SUV_mean_				.009
Median (range)	3.5 (0.2‐13.4)	2.6 (0.2‐12.6)	4.2 (0.4‐13.4)	
SUV_median_				.02
Median (range)	3.0 (0.2‐13.7)	2.5 (0.2‐12.7)	3.7 (0.4‐13.7)	
SUV_min_				.27
Median (range)	0.5 (0.1‐1.4)	0.5 (0.1‐1.2)	0.5 (0.1‐1.4)	
2D diameter of tumor (cm)				.50
Median (range)	3.9 (1.3‐10.5)	3.8 (1.4‐8.6)	3.9 (1.3‐10.5)	
3D diameter of tumor (cm)				.43
Median (range)	4.3 (1.6‐11.7)	4.2 (1.6‐9.8)	4.6 (1.6‐11.7)	
MTV^c^ (cm^3^)				.38
Median (range)	4.6 (0.2‐136.5)	4.3 (0.6‐81.2)	4.7 (0.1‐136.5)	
TLG^d^				.63
Median (range)	14.4 (0.5‐1120.0)	10.6 (0.7‐547.5)	17.0 (0.5‐1120.0)	
Heterogeneity factor				.13
Median (range)	2.2 (0.5‐7.0)	2.2 (0.8‐6.7)	2.4 (0.5‐7.0)	
CEA^e^				>.99
Median (range)	3.3 (0.3‐324.0)	3.1 (0.3‐324.0)	3.3 (0.5‐125.2)	
CYFRA21-1^f^				.47
Median (range)	3.0 (0.9‐78.1)	2.8 (1.1‐28.8)	3.2 (0.9‐78.1)	
NSE^g^				.80
Median (range)	12.0 (7.0‐63.9)	12.1 (7.0‐24.3)	12.0 (7.5‐63.9)	

a Negative or positive refers to PD-L1 expression status.

b SUV: standardized uptake values.

c MTV: metabolic tumor volume.

d TLG: total lesion glucose.

e CEA: carcinoembryonic antigen.

f CYFRA21-1: cytokeratin 19 fragment.

g NSE: neuron-specific enolase.

In the cohort for task A, significant differences were observed between the LNM-positive and LNM-negative groups within the training and validation cohort (n=228), including histopathology (*P*=.04) and histological grade (*P*=.003), visceral pleural invasion (*P*=.002), SUV_max_ (*P*=.01), SUV_mean_ (*P*=.003), SUV_median_ (*P*=.003), metabolic tumor volume (MTV; *P*=.047), and tumor diameters (both 2D and 3D; *P*=.02 and *P*=.03, respectively). These findings align with prior studies establishing links between LNM and factors such as tumor size, pleural invasion, and histopathology [36-38]. Conversely, carcinoembryonic antigen (CEA) and cytokeratin fragment 19 (CYFRA21-1) levels showed no significant association with LNM in our cohort, which contrasts with some previous reports [39,40]. In the temporal validation cohort alone, only the metabolic parameters (SUV_max_, SUV_mean_, and SUV_median_) remained significantly different between LNM-positive and LNM-negative groups (*P*<.05).

For task B, SUV_max_ was the only factor significantly associated with PD-L1 positivity in both the internal validation (*P*=.007) and temporal validation (*P*=.04) cohorts, which is consistent with some previous findings [41].

### Feature Selection Analysis

A total of 7868 radiomics features were extracted from intratumoral and peritumoral regions on PET/CT images.

**Figure 3.**
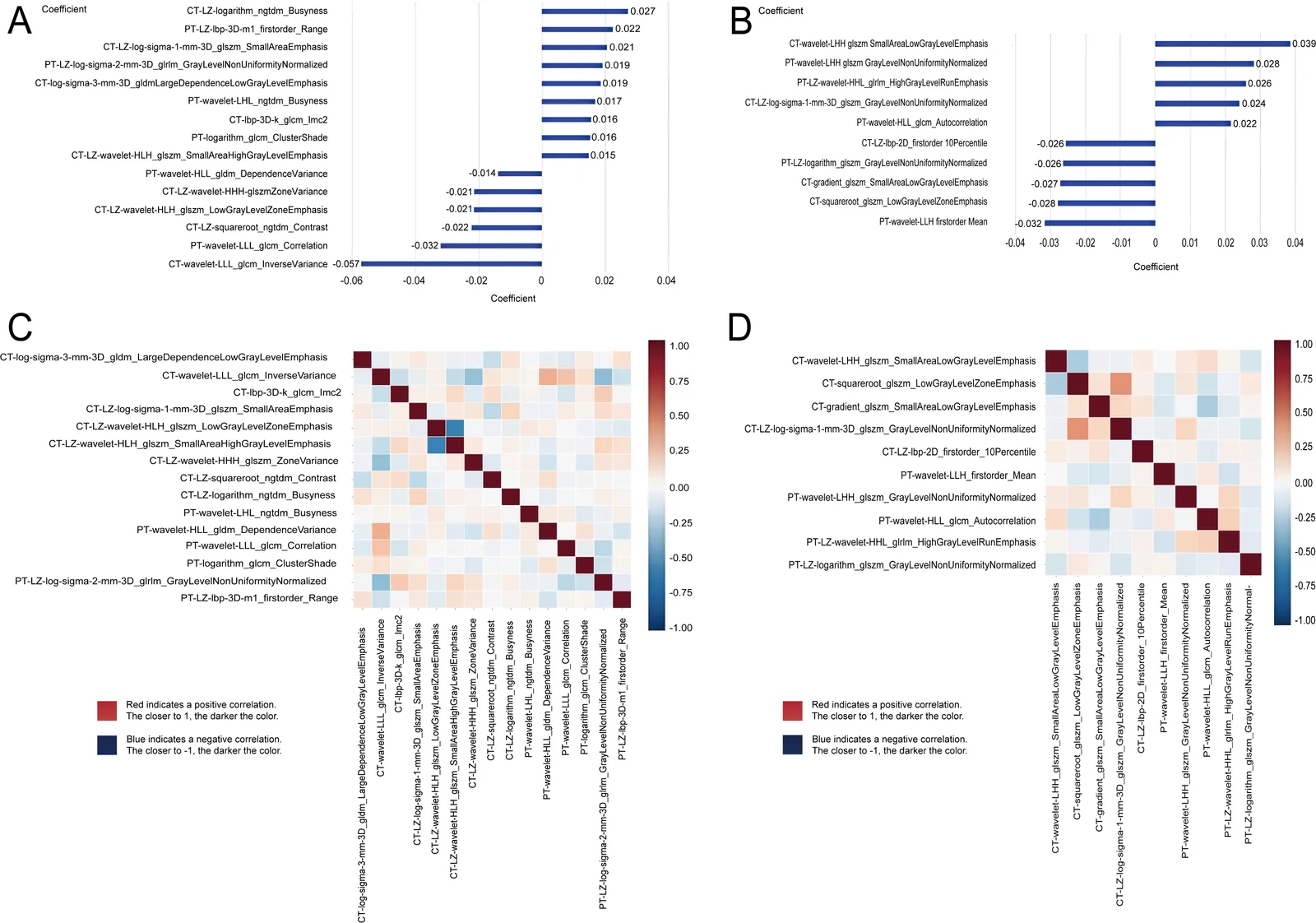
Visualization and correlation of radiomic features for task A and task B. (A) The feature of the positron emission tomography/computed tomography (PET/CT) selected in task A. (B) The feature of the PET/CT selected in task B. (C) The heatmap correlating the features shown in (A). (D) The heatmap correlating the features in (B).

For task A, the selected features were as follows: from CT images, 12 intratumoral features (Figure S2A in Multimedia Appendix 1), 12 peritumoral features (Figure S2B in Multimedia Appendix 1), and 15 intratumoral and peritumoral features (Figure S2C in Multimedia Appendix 1) were selected; from PET images, 12 intratumoral features (Figure S2D in Multimedia Appendix 1), 12 peritumoral features (Figure S2E in Multimedia Appendix 1), and 15 intratumoral and peritumoral features (Figure S2F in Multimedia Appendix 1) were selected; and for PET/CT images, 15 intratumoral and peritumoral features were selected (Figure 3A; Table S2 in Multimedia Appendix 1), with their correlation heatmap presented in Figure 3C. Most extracted features achieved excellent interobserver reproducibility (ICC>0.75), confirming the robustness of our standardized segmentation protocol. All features with ICC of 0.75 or lower were eliminated from the candidate feature set before downstream feature selection.

For task B, the selected features included: 10 intratumoral (Figure S3A in Multimedia Appendix 1), 10 peritumoral (Figure S3B in Multimedia Appendix 1), and 10 combined intratumoral and peritumoral features derived from CT images (Figure S3C in Multimedia Appendix 1); 10 intratumoral (Figure S3D in Multimedia Appendix 1), 10 peritumoral (Figure S3E in Multimedia Appendix 1), and 10 combined intratumoral and peritumoral features extracted from PET images (Figure S3F in Multimedia Appendix 1); as well as 10 combined intratumoral and peritumoral features from integrated PET/CT images (Figure 3B and Table S3 in Multimedia Appendix 1), and their correlation heatmap is illustrated in Figure 3D. Most extracted features achieved ICC values above 0.75.

### Predictive Models and Analysis

In this study, we evaluated the performance of various predictive models for task A and task B. The final clinicopathological models for task A and task B were derived using LASSO-selected feature subsets to avoid multicollinearity. Their specific variables and corresponding regression coefficients are detailed in Tables S4 and S5 in Multimedia Appendix 1.

**Figure 4.**
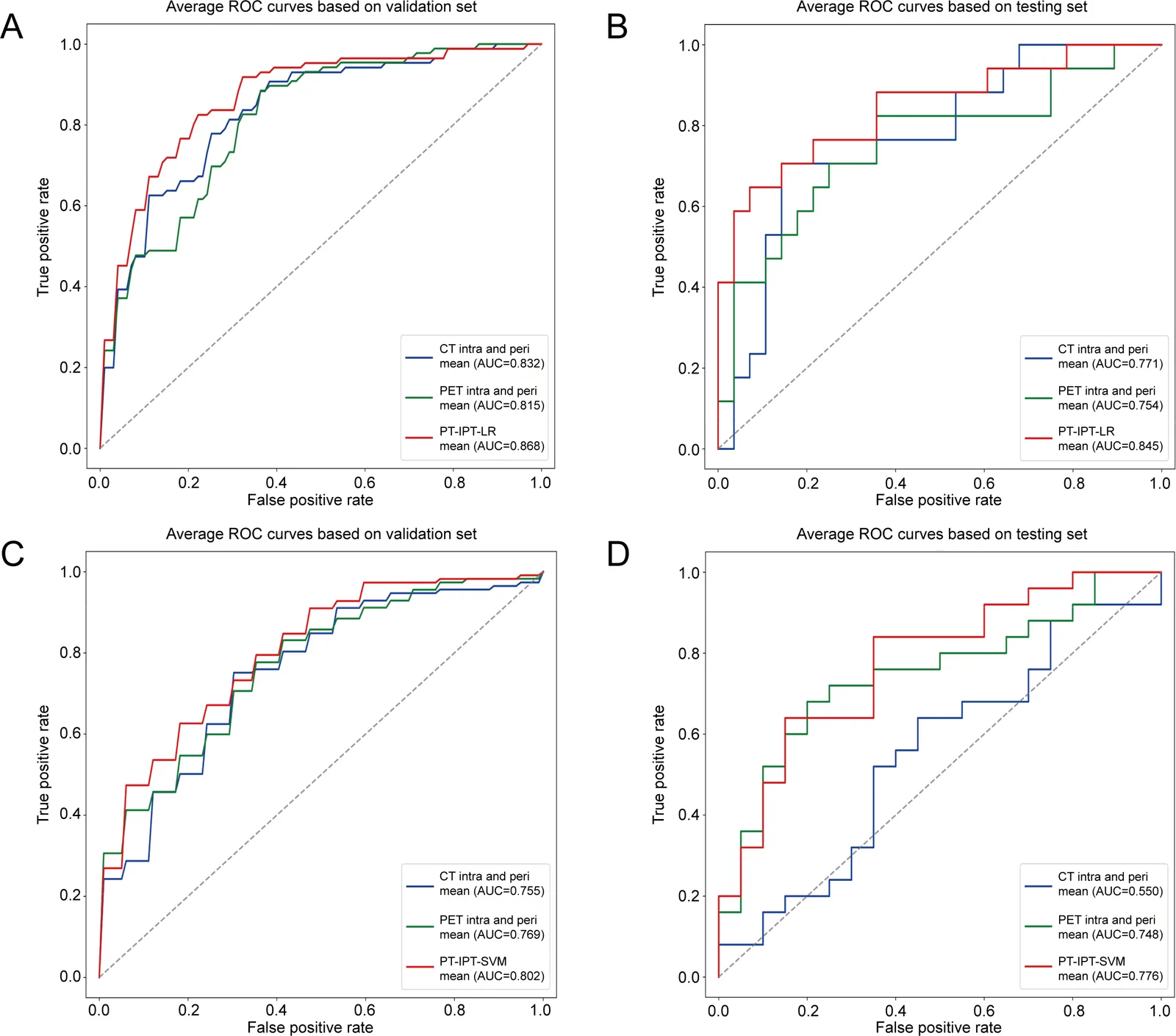
The average receiver operating characteristic (ROC) curves for task A and task B. (A) The average ROC curve on the internal validation set (computed tomography [CT] intratumoral and peritumoral (intra and peri): AUC=0.832; PET intra and peri: AUC=0.815; PT-IPT-LR: AUC=0.868). (B) The average ROC curve on the temporal test set (CT intra and peri: AUC=0.771; PET intra and peri: AUC=0.754; PT-IPT-LR: AUC=0.845). (C) The average ROC curve on the internal validation set (CT intra and peri: AUC=0.755; PET intra and peri: AUC=0.769; PT-IPT-SVM: AUC=0.802). (D) The average ROC curve on the temporal test set (CT intra and peri: AUC=0.550; PET intra and peri: AUC=0.748; PT-IPT-SVM: AUC=0.776). AUC: area under curve.

For task A, on the temporal validation cohort (testing), the PT-IPT-LR model achieved an AUC of 0.845 (95% CI 0.716‐0.973), with a sensitivity of 0.765 (95% CI 0.518‐1.000), a specificity of 0.786 (95% CI 0.602‐0.970), and an accuracy of 0.778 (95% CI 0.630‐0.926; Table 3). Moreover, our model outperformed the diagnostic performance of nuclear medicine physicians, who had a sensitivity of 0.529 and an accuracy of 0.644 on the same cohort. The ROC curves depicting this performance are presented in Figure 4A (validation) and Figure 4B (testing), with further ROC curves available in Figure S4 in Multimedia Appendix 1.

**Table 3.** The predictive model performance for task A^a^.

Model evaluation metrics		CT^b^ intratumoral	PET^c^ intratumoral	CT peritumoral	PET peritumoral	CT intratumoral and peritumoral	PET intratumoral and peritumoral	PT-IPT-LR^d^^,^^e^	Clinicopathology	Physicians
AUC^f^ (95% CI)										
Validation		0.757 (0.612‐0.904)	0.801 (0.673‐0.933)	0.770 (0.633‐0.907)	0.743 (0.597‐0.890)	0.832 (0.712‐0.954)	0.815 (0.691‐0.941)	0.868 (0.765‐0.971)	0.724 (0.658‐0.809)	—^g^
Testing		0.773 (0.624‐0.922)	0.727 (0.568‐0.886)	0.773 (0.624‐0.922)	0.649 (0.479‐0.819)	0.771 (0.621‐0.921)	0.754 (0.601‐0.908)	0.845 (0.716‐0.973)	0.699 (0.551‐0.867)	—
Accuracy										
Validation		0.685	0.698	0.697	0.667	0.780	0.728	0.807	0.656	0.785
Testing		0.756	0.711	0.733	0.622	0.756	0.733	0.778	0.711	0.644
Sensitivity										
Validation		0.686	0.699	0.698	0.640	0.778	0.744	0.813	0.640	0.686
Testing		0.706	0.647	0.765	0.647	0.706	0.647	0.765	0.706	0.529
Specificity										
Validation		0.684	0.698	0.698	0.682	0.781	0.718	0.803	0.666	0.845
Testing		0.786	0.750	0.714	0.607	0.786	0.786	0.786	0.714	0.714
Precision										
Validation		0.562	0.586	0.588	0.555	0.692	0.615	0.717	0.551	0.728
Testing		0.667	0.611	0.619	0.500	0.667	0.647	0.684	0.600	0.529
*F*_1_-score										
Validation		0.615	0.634	0.636	0.591	0.730	0.672	0.760	0.578	0.706
Testing		0.686	0.629	0.684	0.564	0.686	0.647	0.722	0.649	0.529

a Physician validation row values were computed on the full training cohort (n=228). For physician assessment on the testing set (temporal validation cohort), the confusion matrix yielded 9 true positives, 8 false positives, 20 true negatives, and 8 false negatives. For the clinicopathological model on the testing set, the confusion matrix recorded 12 TP, 8 FP, 20 TN, and 5 FN. For the PT-IPT-LR model on the testing set, the confusion matrix showed 13 TP, 6 FP, 22 TN, and 4 FN.

b CT: computed tomography.

c PET: positron emission tomography.

d LR: logistic regression.

e PT-IPT-LR: the logistic regression model based on intratumoral and peritumoral positron emission tomography/computed tomography (PET/CT) signatures.

f AUC: area under curve.

g —: not applicable.

For task B, the PT-IPT-SVM model exhibited strong predictive capabilities (Table 4). During the temporal validation (testing), it achieved an AUC of 0.776 (95% CI 0.641‐0.911), a sensitivity of 0.800 (95% CI 0.609‐0.991), a specificity of 0.650 (95% CI 0.401‐0.899) and an accuracy of 0.733 (95% CI 0.588‐0.879). The model consistently outperformed the clinicopathological benchmark across all evaluation metrics. The ROC curves demonstrating this performance are depicted in Figure 4C (validation) and Figure 4D (testing), with further ROC curves provided in Figure S5 in Multimedia Appendix 1.

To rule out scanner-dependent bias, we further evaluated model performance stratified by scanner manufacturer (Siemens vs UIH). No statistically significant difference in model performance was observed between the 2 scanner subgroups in the temporal testing cohort (task A, *P*=.28, and task B, *P*=.57). These results should be interpreted as compatible with scanner independence rather than as definitive confirmatory evidence. Additional metrics are available in Table S6 in Multimedia Appendix 1.

**Table 4.** The predictive model performance for Task B^a^.

Models		CT intratumoral	PET intratumoral	CT peritumoral	PET peritumoral	CT intratumoral and peritumoral	PET intratumoral and peritumoral	PT-IPT-SVM^b^^,^^c^	Clinicopathology
AUC^d^ (95% CI)									
Validation		0.737 (0.570‐0.904)	0.737 (0.575‐0.900)	0.716 (0.551‐0.882)	0.730 (0.569‐0.894)	0.755 (0.607‐0.904)	0.769 (0.620‐0.921)	0.802 (0.664‐0.942)	0.654 (0.578‐0.750)
Testing		0.608 (0.443‐0.773)	0.716 (0.567‐0.865)	0.478 (0.306‐0.650)	0.758 (0.618‐0.898)	0.550 (0.380‐0.720)	0.748 (0.606‐0.890)	0.776 (0.641‐0.911)	0.648 (0.479‐0.836)
Accuracy									
Validation		0.696	0.681	0.670	0.706	0.691	0.661	0.716	0.617
Testing		0.489	0.644	0.444	0.667	0.578	0.711	0.733	0.622
Sensitivity									
Validation		0.699	0.681	0.670	0.707	0.698	0.673	0.724	0.696
Testing		0.600	0.640	0.400	0.680	0.560	0.720	0.800	0.720
Specificity									
Validation		0.694	0.682	0.671	0.706	0.682	0.674	0.706	0.512
Testing		0.350	0.650	0.500	0.650	0.600	0.700	0.650	0.500
Precision									
Validation		0.750	0.742	0.732	0.762	0.746	0.712	0.772	0.653
Testing		0.536	0.696	0.500	0.708	0.636	0.750	0.741	0.643
*F*_1_-score									
Validation		0.719	0.703	0.691	0.728	0.715	0.679	0.744	0.674
Testing		0.566	0.667	0.444	0.694	0.596	0.735	0.769	0.679

a For the clinicopathological model on the testing set (temporal validation cohort), the confusion matrix recorded 18 true positives, 10 false positives, 10 true negatives, and 7 false negatives. For the PT-IPT-SVM model on the testing set, the confusion matrix showed 20 TP, 7 FP, 13 TN, and 5 FN.

b SVM: support vector machine.

c PT-IPT-SVM: the support vector machine model based on intratumoral and peritumoral positron emission tomography/computed tomography (PET/CT) signatures.

d AUC: area under curve.

### Clinical Utility and Correlation Analysis

**Figure 5.**
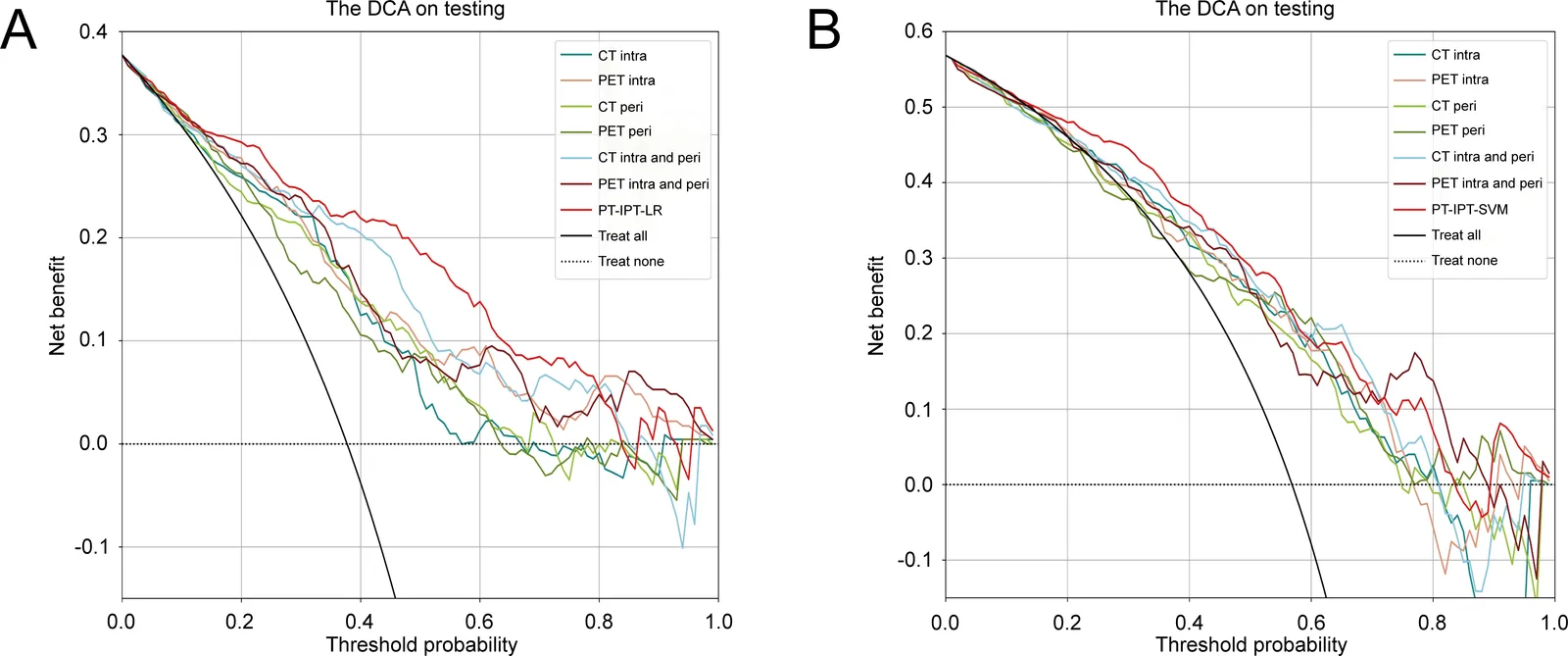
The DCA curves for tasks A and B. (A) The DCA curve for task A. (B) The DCA curve for task B. The horizontal axis represents the threshold probability, which indicates the minimum risk probability that doctors or patients are willing to accept for intervention. The vertical axis is net benefit, reflecting the degree of benefit of model-based decision-making compared with treat all or treat none. A higher value indicates a stronger clinical value of the model. In the plots, different colors represent different feature models. In both subgraphs, the curves of all model combinations are significantly better than treat none, and most are better than treat all. This shows that these radiomics-based models, especially PT-IPT-LR and PT-IPT-SVM, can bring positive net benefits to clinical decision-making and have practical application value. CT: computed tomography; CT intra: the model based on intratumoral CT features; CT intra and peri: the model based on intratumoral and peritumoral CT features; DCA: decision curve analysis; PET: positron emission tomography; PET intra: the model based on intratumoral PET features; CT peri: the model based on peritumoral CT features; PET peri: the model based on peritumoral PET features; PET intra and peri: the model based on intratumoral and peritumoral PET features; PT-IPT-LR: the PET/CT intratumoral and peritumoral logistic regression model; PT-IPT-SVM: the PET/CT intratumoral and peritumoral support vector machine model.

**Figure 6.**
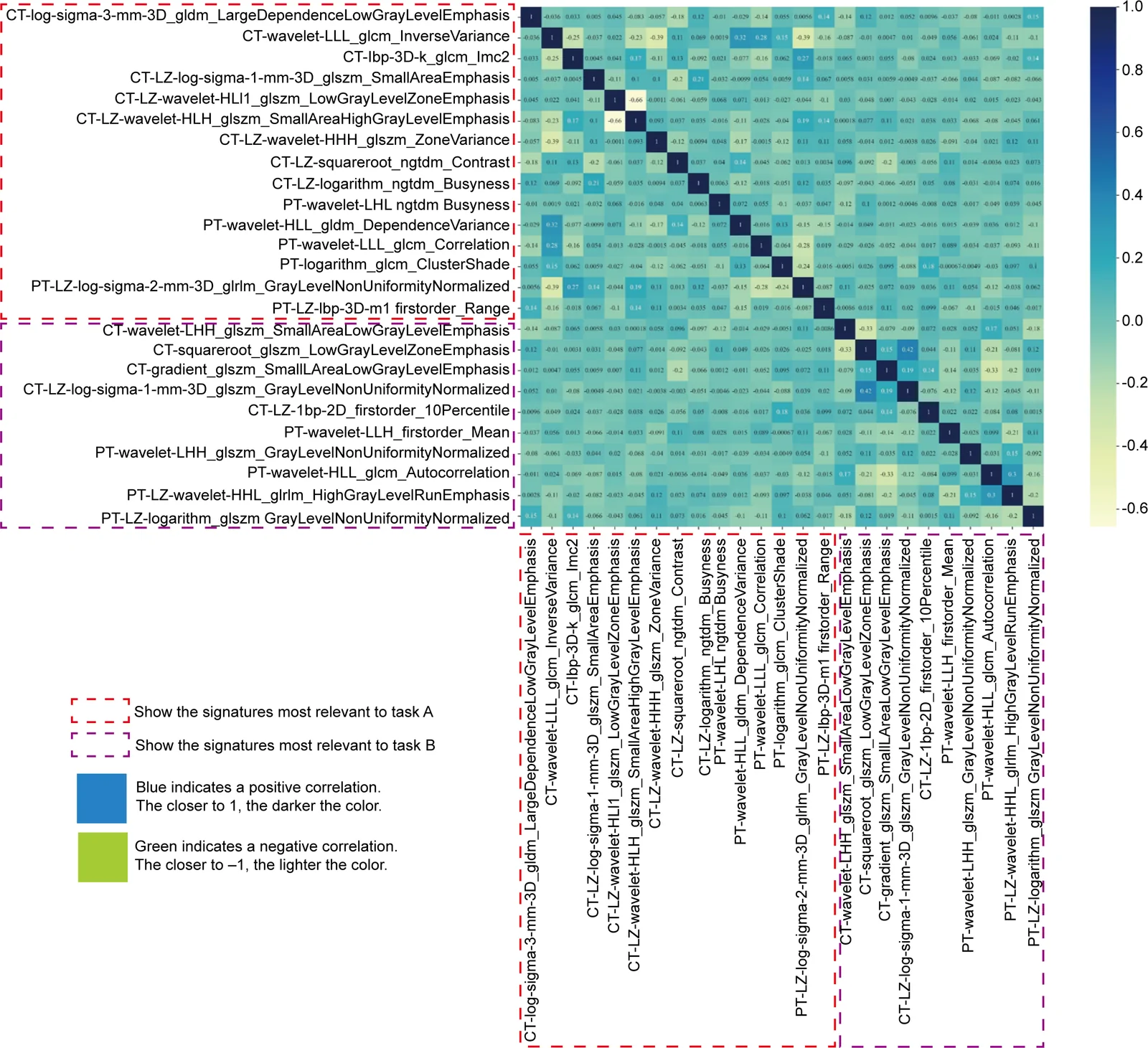
Heatmap of correlation between radiomic features for task A and task B. It shows the association strength and direction of associations between the radiomic features for task A and task B. Both the horizontal and vertical axes list features lists, and the colors represent the correlation coefficients between features. Blue indicates a positive correlation, green indicates a negative correlation, and the lighter the color, the closer the correlation coefficient is to 0, indicating little to no association between the 2 features. The core feature cluster boundaries for task A and task B are clear, with no large-scale overlap, indicating that there are differences in the feature foundations of the 2 task types of tasks.

Integrated PET/CT modality models incorporating intratumoral and peritumoral features showed superior net benefit across threshold probabilities for both task A and task B compared to single-modality models (Figure 5). To verify the correlation of radiomic signatures for LNM and PD-L1 expression, we calculated the average absolute Pearson correlation coefficient between the radiomic signatures of the final task A and task B models. A remarkably low coefficient (*r*=0.051) was obtained, indicating the absence of a significant linear relationship between the 2 signature sets and confirming their statistical independence. Additionally, the heatmap for task A and task B is shown in Figure 6.

## Discussion

### Principal Findings

In this study, we explored the use of a single, routine ^18^F-FDG PET/CT scan to simultaneously predict both LNM and PD-L1 status, thereby offering a new paradigm for achieving a truly “integrated” preoperative assessment. The principal findings can be summarized in 3 points. First, the PT-IPT-LR model, which integrates intratumoral and peritumoral features, demonstrated optimal performance in the LNM prediction task. Second, the PT-IPT-SVM model, also constructed by fusing intratumoral and peritumoral features, showed the best performance in the PD-L1 prediction task. The superior predictive performance of both fused feature model variants substantiates the complementary and synergistic value of intratumoral and peritumoral radiomic features. Analysis of a single feature dimension cannot comprehensively reflect the biological behavior and molecular expression patterns of tumors, while multidimensional feature fusion modeling can better capture the complex biological nature of tumor occurrence and development. Third, through analysis and visualization, no statistically significant correlation was observed between the radiomics features used to predict LNM and those related to PD-L1 expression in tumor tissues.

### Comparison With Prior Work

Our PT-IPT-LR model exhibits outstanding performance in temporal validation for LNM prediction, yielding an AUC value of 0.845. This robust predictive capacity stems from the model’s unique ability to capture the intrinsic biological features of tumors. Unlike previous studies that focused solely on lymph nodes or intratumoral features [16,17], our model innovatively integrates features from both intratumoral and peritumoral regions. The peritumoral region is the frontline of interaction between the tumor and the host microenvironment, and is rich in critical information regarding immune cell infiltration, fibrosis, and angiogenesis [23]. Since LNM is an invasive process, it inevitably leaves traces in the surrounding microenvironment. Consequently, our model can capture these subtle imaging signals beyond the tumor itself, which are associated with invasion and metastasis. This capability reasonably explains why our model significantly surpasses the visual assessment of nuclear medicine physicians, particularly in identifying occult metastases that are metabolically inactive or small in size. This aligns with the findings of Ren et al [42] which suggest that integrated PET/CT radiomics can more effectively evaluate mediastinal and hilar lymph nodes. Our model could serve as a noninvasive triage tool to identify patients at high risk who need invasive staging or extensive nodal assessment, rather than replacing detailed nodal mapping.

Within the domain of immunotherapy prediction, the significance of our PT-IPT-SVM model resides in its direct focus on the core of clinical decision-making, specifically the critical threshold of PD-L1 TPS of 1% or more. We used a PD-L1 TPS threshold of 1% or more for model development, aligning with the standardized definition of PD-L1 positivity used in pivotal combination therapy trials and biomarker reporting. This cutoff provides a broad screening tool to identify the full spectrum of patients potentially eligible for immunotherapy-based regimens. Compared with prior studies linking metabolic parameters to PD-L1 [19,20], our radiomics analysis highlights the critical role of peritumoral features in PD-L1 prediction. This suggests that the tumor immune status is substantially shaped by its microenvironment. Peritumoral radiomics has the potential to capture spatial immune infiltration patterns, information that cannot be obtained from a single biopsy. This likely explains our model’s superior performance over intratumoral-only models (eg, Zhao et al [43], AUC 0.769), and aligns with the concept of a “digital biopsy” emphasized by Monaco et al [44].

One of the most compelling findings of this study is the lack of a significant correlation between the radiomic features associated with LNM and those associated with PD-L1 expression. This fundamentally validates the necessity of developing 2 independent models rather than a single “universal” model. It is worth noting that the feature selection strategies differed between the 2 tasks. For LNM, a hierarchical screening was used to enhance stability against noise from multiple regions of interest, whereas for PD-L1, a direct LASSO approach was adopted to prevent the loss of subtle molecular signals. The former prioritizes feature robustness, while the latter maximizes information retention. The robust temporal validation results for both tasks confirm that the chosen strategies effectively mitigate their respective risks of information loss and overfitting.

DCA further translates this scientific finding into clinical language, confirming that our dual-model strategy can provide a net benefit across threshold probabilities for clinical decision-making. This indicates that during the preoperative planning phase, clinicians can use this tool to simultaneously acquire 2 independent, high-value pieces of decision-support information from the same PET/CT image. Specifically, the PT-IPT-LR model provides guidance on the scope and approach of surgery, whereas the PT-IPT-SVM model offers insights for decisions related to preoperative neoadjuvant immunotherapy or postoperative adjuvant therapy. In some cases of central lung cancer, especially those with obstructive pneumonia or atelectasis, predictions of lymph node metastasis and PD-L1 expression are less reliable (Figure S6 in Multimedia Appendix 1). This phenomenon may be attributed to 2 factors. First, the overlap of CT and PET signals between the tumor and adjacent inflamed or collapsed tissue impedes accurate segmentation and diminishes feature specificity. Second, such complex cases are underrepresented in the training data, a limitation that is exacerbated by potential sampling bias in pathological confirmation. Consequently, clinicians ought to interpret the model predictions for these cases with caution.

Apart from the DCA, the proposed PT-IPT-LR and PT-IPT-SVM models exhibit high compatibility with routine clinical practice. They use standard PET/CT images, eliminating the need for additional scans, and can be applied after routine image interpretation without disrupting the existing workflow. The outputs of the models can be incorporated into electronic medical records to facilitate clinical decision-making in surgical and immunotherapeutic procedures. These models can be integrated with local radiomics platforms, such as uRP in tertiary hospitals, and can also be accessed by primary hospitals via cloud-based application programming interfaces. All radiomics features are based on the open-source *PyRadiomics* library, and feature extraction can be performed using open-source tools or uRP workstations. This process operates efficiently on standard clinical workstations equipped with an Intel Core i5 processor (or equivalent) and a minimum of 8 GB of memory, without the need for high-performance computing. In cases where data from multiple patients need to be analyzed concurrently, the server side can support parallel computing to fulfill clinical batch requirements.

### Limitations and Future Directions

This study has several limitations. First, the validation cohort had a small sample size and a low number of positive events, resulting in a wide 95% CI for the model’s AUC, which limits accurate assessment of its robustness. Related to this, the inherent high dimensionality of radiomic features relative to the cohort size poses a risk of overfitting and spurious correlations. We implemented a rigorous multistep feature selection strategy to mitigate this risk, resulting in parsimonious final models with events per variable ratios of approximately 5.7 for task A and 8.5 for task B. However, because these ratios are only marginally above the commonly cited minimum threshold of 5, the risk of overfitting cannot be completely eliminated. Additionally, the relatively small temporal validation cohort was enrolled at a later time point than the training cohort. This temporal separation, combined with potential variations in patient selection criteria, imaging protocols, and overall case mix across the enrolling centers, may lead to spectrum bias. Therefore, the results should be interpreted with caution. Second, this study performed only preliminary temporal validation. The external universality, exact clinical utility, and broader geographical and technical robustness of the model require further verification. In the future, rigorous external validation should be conducted in large-sample prospective cohorts with balanced baseline characteristics, and multidevice, multiprotocol datasets should be included to more objectively evaluate the actual clinical value of the model in different patient spectra. Third, the relationship between radiomic features and genomic data remains to be explored, and exploring this relationship would help clarify underlying biological mechanisms and enhance interpretability. Fourth, the study cohort has inherent selection bias, as the model was validated only in confirmed surgical candidates. This limits its generalizability to a broader population with NSCLC, including nonsurgical or borderline resectable patients. Future validation in unselected cohorts is needed to establish wider clinical applicability.

### Conclusions

This study establishes the feasibility of a “one-scan, dual-target” assessment for NSCLC, providing noninvasive simultaneous predictions of lymph node metastasis and PD-L1 expression from a single preoperative ^18^F-FDG PET/CT scan. Our validated PT-IPT-LR and PT-IPT-SVM models showed discriminative ability in the study cohort, outperforming conventional diagnostic benchmarks. However, as acknowledged in the limitations, the robustness of these models is constrained by the small sample size and wide CIs. This integrative radiomics framework has the potential to refine preoperative stratification, facilitating more personalized and efficient lung cancer treatment planning, with further validation in larger, multicenter cohorts needed to confirm its robustness and generalizability.

## Supplementary material

10.2196/86835 Multimedia Appendix 1 Methodological details and comprehensive supplementary results.

## Abbreviations

^18^F-FDG PET/CT: ^18^F-fluoro-2-deoxy-D-glucose positron emission tomography/computed tomography
AUC: area under the curve
CEA: carcinoembryonic antigen
CT: computed tomography
CYFRA21-1: cytokeratin fragment 19
EGFR: epidermal growth factor receptor
IHC: immunohistochemistry
LASSO: least absolute shrinkage and selection operator
LN: lymph node
LNM: lymph node metastasis
LoG: Laplacian of Gaussian
MTV: metabolic tumor volume
NSCLC: non-small cell lung cancer
OSEM: ordered-subsets expectation maximization
PD-L1: programmed death-ligand 1
PET: positron emission tomography
ROC: receiver operating characteristic
SUV: standardized uptake values
TNM: tumor node metastasis
TPS: tumor proportion score
UIH: United Imaging Healthcare
