# Supplementary material for: Integrating Lymph Node Metastasis and Programmed Death-Ligand 1 Prediction in Non–Small Cell Lung Cancer From a Single PET/CT Scan: Multicenter Radiomics Study
Source: JMIR Med Inform. 2026 Sep 9;14:e86835. doi: 10.2196/86835 (PMC13557200; doi:10.2196/86835)
Supplement: Supplementary file 1 [file medinform-v14-e86835-s001.docx]

**Integrating Lymph Node Metastasis and PD-L1 Prediction in NSCLC from a Single PET/CT Scan: A Multicenter Radiomics Study**

This appendix provides supporting methodological details and comprehensive supplementary results. It includes the demographic and clinical characteristics of the temporal validation cohort (Table S1); numerical coefficients, feature weights, and intercepts for the final radiomics models (Tables S2–S3) and the clinicopathological benchmark models (Tables S4–S5); and a scanner-stratified performance analysis (Table S6). Additionally, it details the model hyperparameters (Supplementary S1) and illustrates the feature selection workflow (Figure S1), specific feature coefficients for both prediction tasks (Figures S2–S3), ROC curves of individual sub-models on the validation and test sets (Figures S4–S5), and a representative mispredicted case with corresponding PET/CT images (Figure S6).

**Supplementary Table 1.** The demographic and clinical characteristics of the temporal validation cohort. The P-value represents the comparison between groups. LNM, lymph node metastasis; MTV, metabolic tumor volume; TLG, total lesion glucose; CEA, carcinoembryonic antigen; CYFRA21-1, cytokeratin 19 fragment; NSE, neuron-specific enolase; SUV, standardized uptake value.

| Clinical characteristic | Task A (LNM status) | | | | Task B (PD-L1 status) | | | |
| --- | --- | --- | --- | --- | --- | --- | --- | --- |
|  | All | No LNM | LNM | P-value | All | Negative | Positive | P*-*value |
| Number  (% of total) | 45 (100.0) | 28 (62.2) | 17 (37.8) |  | 45 (100.0) | 20 (44.4) | 25 (55.6) |  |
| Age (years) |  |  |  | .186 |  |  |  | .064 |
| Mean ± SD (range) | 61.3 ± 11.8 (27–81) | 63.9 ± 12.0 (27–77) | 57 ± 10.7 (48–81) |  | 61.3 ± 11.8 (27–81) | 63.9 ± 11.1 (37–81) | 59.2 ± 11.3 (27–74) |  |
| Sex, n (%) |  |  |  | .189 |  |  |  | .940 |
| Female | 16 (35.6) | 12 (42.9) | 4 (23.5) |  | 16 (35.6) | 7 (35.0) | 9 (36.0) |  |
| Male | 29 (64.4) | 16 (57.1) | 13 (76.5) |  | 29 (64.4) | 13 (65.0) | 16 (64.0) |  |
| Histopathology, n (%) | |  |  | .936 |  |  |  | .633 |
| Squamous cell carcinoma | 10 (22.2) | 6 (21.4) | 4 (23.5) |  | 10 (22.2) | 5 (25.0) | 5 (20.0) |  |
| Adenocarcinoma | 32 (71.1) | 20 (71.4) | 12 (70.6) |  | 32 (71.1) | 13 (65.0) | 19 (76.0) |  |
| Others | 3 (6.7) | 2 (7.1) | 1 (5.9) |  | 3 (6.7) | 2 (10.0) | 1 (4.0) |  |
| Grade of histology, n (%) | | |  | .106 |  |  |  | .091 |
| Poorly | 15 (33.3) | 9 (32.1) | 6 (35.3) |  | 15 (33.3) | 3 (15.0) | 12 (48.0) |  |
| Moderately- poorly | 14 (31.1) | 4 (14.3) | 10 (58.8) |  | 14 (31.1) | 7 (35.0) | 7 (28.0) |  |
| Moderately | 13 (28.9) | 12 (42.9) | 1 (5.9) |  | 13 (28.9) | 7 (35.0) | 6 (24.0) |  |
| Highly- moderately | 2 (4.4) | 2 (7.1) | 0 (0.0) |  | 2 (4.4) | 2 (10.0) | 0 (0.0) |  |
| Highly | 1 (2.2) | 1 (3.6) | 0 (0.0) |  | 1 (2.2) | 1 (5.0) | 0 (0.0) |  |
| Unknown | 0 (0.0) | 0 (0.0) | 0 (0.0) |  | 0 (0.0) | 0 (0.0) | 0 (0.0) |  |
| Primary tumor location, n (%) | | |  | .809 |  |  |  | .920 |
| Right upper lobe | 10 (22.2) | 5 (17.9) | 5 (29.4) |  | 10 (22.2) | 5 (25.0) | 5 (20.0) |  |
| Left upper lobe | 14 (31.1) | 10 (35.7) | 4 (23.5) |  | 14 (31.1) | 5 (25.0) | 9 (36.0) |  |
| Left lower lobe | 4 (8.9) | 3 (10.7) | 1 (5.9) |  | 4 (8.9) | 2 (10.0) | 2 (8.0) |  |
| Right lower lobe | 14 (31.1) | 8 (28.6) | 6 (35.3) |  | 14 (31.1) | 7 (35.0) | 7 (28.0) |  |
| Right middle lobe | 3 (6.7) | 2 (7.1) | 1 (5.9) |  | 3 (6.7) | 1 (5.0) | 2 (8.0) |  |
| Imaging classification, n (%) | | |  | .447 |  |  |  | 1.000 |
| Central | 9 (20.0) | 7 (25.0) | 2 (11.8) |  | 9 (20.0) | 4 (20.0) | 5 (20.0) |  |
| Peripheral | 36 (80.0) | 21 (75.0) | 15 (88.2) |  | 36 (80.0) | 16 (80.0) | 20 (80.0) |  |
| Central with peripheral | 0 (0.0) | 0 (0.0) | 0 (0.0) |  | 0 (0.0) | 0 (0.0) | 0 (0.0) |  |
| Visceral pleural invasion, n (%) | | |  | .063 |  |  |  | .260 |
| Yes | 9 (20.0) | 3 (10.7) | 6 (35.3) |  | 9 (20.0) | 2 (10.0) | 7 (28.0) |  |
| No | 33 (73.3) | 23 (82.1) | 10 (58.8) |  | 33 (73.3) | 16 (80.0) | 17 (68.0) |  |
| Unknown | 3 (6.7) | 2 (7.1) | 1 (5.9) |  | 3 (6.7) | 2 (10.0) | 1 (4.0) |  |
| Volume of tumor (cm^3^) | | |  | .795 |  |  |  | .298 |
| Median (range) | 14.8 (0.8–592.1) | 6.4 (0.8–227.8) | 16.4 (2.9–592.1) |  | 14.8 (0.8–592.1) | 17.7 (0.8–592.1) | 14.3 (1.2–85.5) |  |
| SUV_max_ |  |  |  | .020 |  |  |  | .042 |
| Median (range) | 10.9 (1.7–39.5) | 5.3 (1.7–20.7) | 12.6 (4.1–39.5) |  | 10.9 (1.7–39.5) | 6.1 (1.7–22.0) | 12.3 (4.0–39.5) |  |
| SUV_mean_ |  |  |  | .006 |  |  |  | .063 |
| Median (range) | 3.9 (1.0–12.6) | 2.3 (1.0–8.2) | 4.8 (1.8–12.6) |  | 3.9 (1.0–12.6) | 2.5 (1.0–9.7) | 5.2 (1.7–12.6) |  |
| SUV_median_ |  |  |  | .006 |  |  |  | .078 |
| Median (range) | 3.5 (0.9–45.1) | 3.2 (0.9–36.2) | 7.4 (1.6–45.1) |  | 3.5 (0.9–45.1) | 2.3 (0.9–10.6) | 4.1 (1.5–45.1) |  |
| SUV_min_ |  |  |  | .574 |  |  |  | .535 |
| Median (range) | 0.5 (0.1–1.2) | 0.5 (0.1–1.2) | 0.4 (0.1–1.0) |  | 0.5 (0.1–1.2) | 0.4 (0.1–0.7) | 0.6 (0.2–1.2) |  |
| Two-dimensional diameter of tumor (cm) | | | | .422 |  |  |  | .098 |
| Median (range) | 3.9 (1.4–12.8) | 3.5 (1.4–10.5) | 4.2 (2.2–12.8) |  | 3.9 (1.4–12.8) | 4.1 (1.4–12.8) | 3.9 (1.7–6.8) |  |
| Three-dimensional diameter of tumor (cm) | | | | .325 |  |  |  | .100 |
| Median (range) | 4.7 (1.7–15.4) | 3.6 (1.7–11.7) | 4.7 (2.5–15.4) |  | 4.7 (1.7–15.4) | 5.1 (1.7–15.4) | 4.6 (1.8–8.8) |  |
| MTV (cm^3^) |  |  |  | .335 |  |  |  | .585 |
| Median (range) | 7.2 (0.5–129.0) | 3.2 (0.5–36.2) | 7.4 (1.6–129.0) |  | 7.2 (0.5–129.0) | 7.7 (0.5–129.0) | 6.6 (0.5–44.5) |  |
| TLG |  |  |  | .119 |  |  |  | .334 |
| Median (range) | 24.6 (0.8–1226.0) | 5.8 (0.8–296.7) | 32.4 (3.4–1226.0) |  | 24.6 (0.8–1226.0) | 16.6 (0.8–1226.0) | 35.2 (1.4–249.8) |  |
| Heterogeneity factor | |  |  | .422 |  |  |  | .097 |
| Median (range) | 2.3 (1.1–4.0) | 2.2 (1.1–4.0) | 2.4 (1.7–4.0) |  | 2.3 (1.1–4.0) | 2.3 (1.1–4.0) | 2.3 (1.8–3.4) |  |
| CEA |  |  |  | .628 |  |  |  | .404 |
| Median (range) | 2.4 (0.9–97.2) | 2.2 (0.9–97.2) | 4.0 (1.2–30.1) |  | 2.4 (0.9–97.2) | 2.5 (1.1–42.1) | 2.4 (0.9–97.2) |  |
| CYFRA21-1 |  |  |  | .289 |  |  |  | .737 |
| Median (range) | 2.8 (1.3–189.0) | 3.0 (1.3–14.8) | 2.7 (1.4–189.0) |  | 2.8 (1.3–189.0) | 2.5 (1.7–189.0) | 2.9 (1.3–9.7) |  |
| NSE |  |  |  | .107 |  |  |  | .676 |
| Median (range) | 12.4 (7.0–39.5) | 12.7 (7.0–18.8) | 14.5 (8.0–39.5) |  | 12.4 (7.0–39.5) | 13.7 (7.0–39.5) | 12.3 (8.3–27.9) |  |

Note: Negative/Positive refers to PD-L1 expression status.

**Supplementary Table 2.** The numerical feature weights, coefficients, and intercept terms of PT-IPT-LR (Task A).

| Features of Task A | Coefficient |
| --- | --- |
| CT-log-sigma-3-mm-3D_gldm_LargeDependenceLowGrayLevelEmphasis | 0.019 |
| CT-wavelet-LLL_glcm_InverseVariance | -0.057 |
| CT-lbp-3D-k_glcm_Imc2 | 0.016 |
| CT-LZ-log-sigma-1-mm-3D_glszm_SmallAreaEmphasis | 0.021 |
| CT-LZ-wavelet-HLH_glszm_LowGrayLevelZoneEmphasis | -0.021 |
| CT-LZ-wavelet-HLH_glszm_SmallAreaHighGrayLevelEmphasis | 0.015 |
| CT-LZ-wavelet-HHH_glszm_ZoneVariance | -0.021 |
| CT-LZ-squareroot_ngtdm_Contrast | -0.022 |
| CT-LZ-logarithm_ngtdm_Busyness | 0.027 |
| PT-wavelet-LHL_ngtdm_Busyness | 0.017 |
| PT-wavelet-HLL_gldm_DependenceVariance | -0.014 |
| PT-wavelet-LLL_glcm_Correlation | -0.032 |
| PT-logarithm_glcm_ClusterShade | 0.016 |
| PT-LZ-log-sigma-2-mm-3D_glrlm_GrayLevelNonUniformityNormalized | 0.019 |
| PT-LZ-lbp-3D-m1_firstorder_Range | 0.022 |
| Intercept | -0.501 |

Note: The logistic regression intercept equals the log-odds of the positive class under Z-score standardization of predictors.

**Supplementary Table 3.** Feature selection weights and Linear SVM surrogate model coefficients for the PT-IPT-SVM model (Task B).

| Features of Task B | LASSO Selection Weight | Linear SVM Surrogate Coefficient | StandardScaler (Mean) | StandardScaler (Standard Deviation) |
| --- | --- | --- | --- | --- |
| CT-wavelet-LHH_glszm_SmallAreaLowGrayLevelEmphasis | 0.039 | 0.339 | 0.323 | 0.155 |
| CT-squareroot_glszm_LowGrayLevelZoneEmphasis | -0.028 | -0.480 | 0.204 | 0.144 |
| CT-gradient_glszm_SmallAreaLowGrayLevelEmphasis | -0.027 | -0.385 | 0.175 | 0.080 |
| CT-LZ-log-sigma-1-mm-3D_glszm_GrayLevelNonUniformityNormalized | 0.024 | 0.667 | 0.278 | 0.037 |
| CT-LZ-lbp-2D_firstorder_10Percentile | -0.026 | -0.459 | 2.020 | 0.200 |
| PT-wavelet-LLH_firstorder_Mean | -0.032 | -0.582 | -0.033 | 0.025 |
| PT-wavelet-LHH_glszm_GrayLevelNonUniformityNormalized | 0.028 | 0.335 | 0.553 | 0.062 |
| PT-wavelet-HLL_glcm_Autocorrelation | 0.022 | 0.327 | 2.130 | 0.227 |
| PT-LZ-wavelet-HHL_glrlm_HighGrayLevelRunEmphasis | 0.026 | 0.206 | 2.495 | 0.015 |
| PT-LZ-logarithm_glszm_GrayLevelNonUniformityNormalized | -0.026 | -0.175 | 0.756 | 0.228 |
| Intercept | 0.276† | 0.367‡ | – | – |

Note:†LASSO/LR intercept equals the log-odds of the positive class under Z-score standardization of predictors. ‡ intercept is in the decision function space; converting to probability: σ(0.367) ≈ 0.591. RBF-SVM hyperparameters: kernel = RBF, C = 1.0, gamma = 0.01. Linear SVM surrogate: kernel = linear, C = 1.0, random state = 42. All features were Z-score standardized before model training.

**Supplementary Table 4.** Variables and coefficients of the final clinicopathological models for Task A.

| Clinical/Pathological/Imaging Variable | Coefficient |
| --- | --- |
| Pathology (histological type) | 0.024 |
| SUV_mean_ | 0.035 |
| Differentiation | -0.039 |
| Visceral pleura affected | 0.053 |
| Intercept | -0.501 |

Note: The logistic regression intercept equals the log-odds of the positive class under Z-score standardization of predictors.

**Supplementary Table 5.** Variables and coefficients of the final clinicopathological models for Task B.

| Clinical/Pathological/Imaging Variable | Coefficient |
| --- | --- |
| Visceral pleura affected | 0.037 |
| SUV_max_ | 0.059 |
| Intercept | 0.276 |

Note: The logistic regression intercept equals the log-odds of the positive class under Z-score standardization of predictors.

**Supplementary Table 6.** Scanner-stratified performance analysis. LNM, lymph node metastasis; AUC, area under curve; CI, confidence interval; UIH, United Imaging Healthcare.

| Task | Scanner Manufacturer | Sample Size | AUC (95% CI) | P-value |
| --- | --- | --- | --- | --- |
| LNM prediction | Siemens | 23 | 0.850  (0.641–0.968) | .276 |
|  | UIH | 22 | 0.750  (0.519–0.917) |  |
| PD-L1 prediction | Siemens | 23 | 0.790  (0.581–0.943) | .571 |
|  | UIH | 22 | 0.750  (0.528–0.912) |  |

**Supplementary S1. Model Development and Evaluation**

The final hyperparameters for the PT-IPT-LR model consist of a C value of 1.5, an L2 penalty, a balanced class weight, and a tolerance of 1e−05. The final hyperparameters for the PT-IPT-SVM model consist of a C value of 1.0, a gamma of 0.01, an RBF kernel, and class_weight = None. The linear SVM was configured as SVC (kernel = ‘linear’, C = 1.0, random state = 42) with all input features preprocessed via Z-score standardization (StandardScaler). Importantly, the above linear-SVM hyperparameters are provided only as a reproducible surrogate reference rather than parameters of our final nonlinear RBF-SVM model.

**Supplementary Fig. 1.** Workflow of the multi-step feature selection strategy for radiomic features. Radiomic features extracted from intratumoral and peritumoral regions across CT and PET modalities were subjected to variance thresholding, correlation filtering, and LASSO regression to select the final predictive signatures. Clinical variables were not included in this radiomics pipeline and were used exclusively to construct the separate clinicopathological benchmark model. Task A employed a hierarchical multi-step approach with preliminary modality-specific and region-specific screening before final LASSO selection, while Task B applied LASSO directly to the entire raw radiomic feature pool to avoid information loss for this molecular phenotype.

**Supplementary Fig. 2.** Feature selection details for Task A (CT/PET). The vertical axis of these subgraphs (**a-f**) represents the names of radiomic features corresponding to different modalities and different processing methods, and the horizontal axis represents the coefficients of the features in the model (reflecting the magnitude and direction of their weights). (**a**), (**b**) and (**c**) show the intratumoral, peritumoral, and combined intratumoral and peritumoral CT features. (**d**), (**e**) and (**f**) show the intratumoral, peritumoral, and combined intratumoral and peritumoral PET features.

**Supplementary Fig. 3.** Feature selection details for Task B (CT/PET). The vertical axis of these subgraphs (**a-f**) represents the names of radiomic features corresponding to different modalities and different processing methods, and the horizontal axis represents the coefficients of the features in the model (reflecting the magnitude and direction of their weights). (**a**), (**b**) and (**c**) show the intratumoral, peritumoral, and combined intratumoral and peritumoral CT features. (**d**), (**e**) and (**f**) show the intratumoral, peritumoral, and combined intratumoral and peritumoral PET features.

**Supplementary Fig. 4.** The average ROC curves of individual models for Task A. (**a**) and (**b**) represent CT intratumoral ROC curves on validation set and test set. (**c**) and (**d**) represent CT peritumoral ROC curves on validation set and test set. **(e)** and **(f)** represent PET intratumoral ROC curves on validation set and test set. **(g)** and **(h)** represent PET peritumoral ROC curves on validation set and test set.

**Supplementary Fig. 5.** The average ROC curves of individual models for Task B. (**a**) and (**b**) represent CT intratumoral ROC curves on validation set and test set. (**c**) and (**d**) represent CT peritumoral ROC curves on validation set and test set. (**e**) and (**f**) represent PET intratumoral ROC curves on validation set and test set. (**g**) and (**h**) represent PET peritumoral ROC curves on validation set and test set.

**Supplementary Fig. 6**. Representative mispredicted case for our predictive models. A patient with lung squamous cell carcinoma, having a mass in the right lower lobe of the lung along with multiple patchy shadows around it. (a), (b), and (c) display axial PET images. (d), (e), and (f) show lung-window CT scans. (g), (h), and (i) present fused PET/CT images. Red arrows mark the primary tumor while blue arrows indicate surrounding inflammatory patchy shadows. Postoperative pathological confirmation revealed negative lymph node metastasis and a PD-L1 tumor proportion score of 95%. The model incorrectly predicted lymph node metastasis presence and low PD-L1 expression, conflicting with the gold-standard pathological findings.
